# Clinical and radiographic assessment of mineral trioxide aggregate with platelet rich fibrin as pulp capping biomaterials: a 12-month randomized trial

**DOI:** 10.1038/s41598-025-96056-w

**Published:** 2025-04-15

**Authors:** Rahma Ahmed Ibrahem Hafiz Abuhashema, Mona El Saied Essa, Shereen Hafez Ibrahim, Omaima Mohamed Safwat

**Affiliations:** 1 Conservative Dentistry Department, Faculty of Dentistry, Cairo University, Cairo, Egypt; 2 Conservative Dentistry Department, Faculty of Dentistry, Beni-Suef University, Beni-Suef, Egypt

**Keywords:** Pulp exposure, dentin Bridge formation, Direct pulp capping, Mineral trioxide aggregate, Platelet rich fibrin, Success rate, CBCT, Pulp conservation, Mineral trioxide aggregate

## Abstract

This study aimed to comparatively assess the clinical success and radiographic regenerative dentin formation of Platelet Rich Fibrin (PRF) and mineral trioxide aggregate (MTA) when used as direct pulp capping agents. This double-blinded two parallel armed randomized controlled clinical trial comprised the allocation of 108 patients with traumatically exposed dental pulp during the management of deep carious lesions by undergraduate students after fulfilling inclusion and exclusion criteria. Patients were randomized into two groups (*n* = 54 in each group) using computer-generated simple randomization, wherein one group Platelet Rich Fibrin (PRF) was prepared from patients’ blood samples and applied directly over exposed pulp followed by MTA application and in the other group MTA was applied directly over pulp exposure. In both groups, cavities were restored with resin-modified glass ionomer liner and resin composite restoration. The overall success of treatment was calculated at 6 and 12 months after assessing pulp sensibility, history of pain, tenderness on percussion and the existence of any periapical pathosis using in periapical radiographs. Moreover CBCT was used at 12 months to determine the presence or absence of dentin bridge as a secondary outcome. After 12 months follow-up, there was no statistically significant difference in overall success of pulp capping in both groups. As the both groups showed 92.59% success rate. CBCT evaluation of dentin bridge formation by Platelet Rich Fibrin (PRF) demonstrated a significantly higher percentage than that formed in cases treated with MTA alone (*p* < 0.001). Direct pulp capping with Platelet Rich Fibrin (PRF) exhibited a clinical and radiographic success rate comparable to that of MTA. Platelet Rich Fibrin (PRF) can be implemented as a direct pulp capping agent in forthcoming clinical applications.

## Introduction

Preservation of pulp vitality with biologically based management techniques is considered the cornerstone while managing deep caries^1^. The tooth response to injury represents a complex interplay between injury, defense and regenerative processes^2^. Vital pulp therapy such as direct pulp capping is a procedure geared toward maintaining pulp vitality in teeth with reversible pulp inflammation through hard tissue healing and repair of the opened exposure^3^. Thus reducing the intervention and preserving pulp developmental, defensive and proprioceptive functions^4,5^ along with retaining tooth sensibility, innervation and immunological response, as well as the ability of dentinogenesis and even root development^6,7^. Cost-effectiveness analysis has shown vital pulp therapy to be superior to root canal treatment^8^.

Vital pulp therapy can be performed with a wide variety of biomaterials; however, according to systematic reviews, identifying the best pulp capping agent for each clinical case is still ambiguous and a problematic issue owing to multiple studies showing significant potential for bias and poor methodological quality beside brief follow-up durations that are inadequate for assessing long-term effects of different direct pulp capping materials^7,9–11^. Among many capping materials, the frequently used ones are calcium hydroxide and mineral trioxide aggregate.

Calcium hydroxide has been regarded as the “gold standard” for direct pulp capping materials for numerous decades^12,13^. Its remarkable antimicrobial capabilities have contributed to this popularity. It has a proven history of clinical efficacy as a direct pulp-capping agent for durations lasting up to 10 years; nevertheless, diminished success rates have been observed in studies particularly when dental students executed the procedures. It can be explained by the fact that Ca(OH)_2_ lacks adhesiveness to dentin, it is exceedingly soluble, dissolves with time, and eventually disappears compromising the effective seal. Furthermore, the produced dentin wall was discovered to have tunnel flaws acting as a passageway for the flow of microbes^14,15^. Its Clinical success rates in pulp capping procedures fluctuate significantly among studies, ranging from 13–97.8%^16^.

MTA has emerged as one of the most extensively researched materials. It possesses numerous advantageous characteristics such as low solubility, radiopacity, bioactivity, hydrophilicity, and sealing ability^17,18^, besides its capacity to facilitate the release of bioactive dentin matrix proteins. Studies showed that the inflammation induced by calcium silicate materials is merely momentary, less intense, and less extensive with negligible necrosis of pulp tissue compared to that caused by Ca(OH)_2_^19,20^. Additionally, MTA was speculated to have an antimicrobial effect against *Streptococcus mutans*, *Streptococcus sanguis* and *E. Faecalis*. that is primarily attributed to their elevated alkalinity and calcium ion-releasing capability^19,21,22^.

Several studies have reported high success rates of vital pulp therapy with MTA, where the survival analysis in a multicenter clinical trial by **Kundzina et al.** in **2017** with a 3-year follow-up showed an 85% cumulative survival rate of pulps^23^. According to a systematic review and meta-analysis by **Paula et al.**, The success rate of direct pulp capping using MTA cement varies from 80.3 to 100% (average of 91.1%), whereas dentin bridge development ranges from 33.3 to 100% (average of 74%)^24^.

Although hydraulic calcium silicate cement is highly suggested by European Society of Endodontics to be the sealing material of choice in direct pup capping procedures^25^, Nevertheless, MTA possesses many drawbacks as well, including its propensity to discolor teeth, exhibiting elevated initial cytotoxicity since the mixed MTA is surrounded by an elevated alkalinity persisting for eight weeks having a cytotoxic impact on macrophages and fibroblasts and influencing cell growth^26^, and the inclusion of hazardous substances and trace elements such as arsenic^27–30^ and aluminium phase of the Portland cement as it forms a potential threat of Alzheimer’s disease when comes in direct contact with human tissues^21,28^.

Thereby, there is a necessity for biologically derived autologous materials to mitigate the negative effects of MTA, diminish pulpal inflammation, and expedite the healing process^31^. Platelet-rich fibrin (PRF) is a second-generation platelet concentrate and autologous source of platelets without additives or biochemical handling of blood that is enriched with several growth factors that influence and direct the process of reparative dentinogenesis^2,32,33^. It is more biocompatible toward pulp, and hence it elicits minimal or nil inflammatory response when placed directly over the amputated pulp^34,35^. It has the potential to support pulpal healing by moderating pulpal inflammation through the release of healing cytokines^36^and yielded promising clinical outcomes with significant dentin bridge formation^37^.

A systematic review by ***Noor Mohamed et al.***, found that platelet concentrate showed 88–100% success in pulpotomy compared to control groups (Ca(OH)_2_ and MTA) which showed 80–96%^38^ **.** ***Smoczer et al.***,*** in 2023*** reported that the combination of Platelet Rich Fibrin (PRF) and calcium-based silicate materials had no negative impact on Stem cell viability or migration^39^. However, high-quality randomized clinical trials are highly advocated due to the paucity of studies^38^.

Recently, with the paradigm shift in regenerative dentistry to promote and foster the pulp healing process using acellular approach triggering odontoblast-like cells to differentiate and produce tertiary dentin in the site of exposure^40,41^, with the limited availability of research in this area, this study aimed to assess if the application of platelet rich fibrin in direct pulp capping as a biological scaffold has a similar success rate and regenerative dentin formation to mineral trioxide aggregate applied alone. The null hypothesis tested was that application of Platelet Rich Fibrin (PRF) prior to MTA in direct pulp capping therapies would have a similar success rate and radiographic regenerative dentin formation to the application of MTA alone.

## Materials and methods

### Study setting, design and registration

This study was a randomized and two-parallel armed clinical trial with an equal allocation ratio of 1:1. The protocol of the current study was registered in (www.clinicaltrails.gov) on (28/07/2020), with an identification number NCT04488679. All the study logistical and clinical procedures were approved by the research ethics committee of the faculty of dentistry, Cairo University (Approval no. 13.9.20) which was in accordance with the ethical standard of Helsinki. This clinical study took place from 2021 to 2024 in the outpatient clinic of Cairo University (Faculty of Dentistry, Conservative Dentistry Department).

### Sample size calculation

Sample size calculation was performed using R statistical analysis software version 4.3.2 for Windows. A power analysis was designed to have adequate power to apply a two-sided statistical test of the null hypothesis that there is no difference would be found between different tested groups regarding success rate. By adopting an alpha (α) level of (0.05) and a beta (β) level of (0.2) (i.e. power = 80%) and an effect size (ɷ) of (0.297) calculated based on the results of a previous study^42^; the total required sample size (n) was found to be (90) cases (i.e., 45 cases per group). Sample size was increased by (20%) to compensate for possible dropouts during different follow up intervals to be (108) cases (54 cases per group).

### Enrolment of the patients

Patients with exposed pulp during carious lesion operative management by undergraduate students in the faculty of dentistry, Cairo University were enrolled after screening whether they met the eligibility criteria or not. Medical and dental history were checked. Clinical and intraoral assessment in terms of pain, tenderness on percussion, periodontal status, and restorability of the tooth were ascertained preoperatively. Intraoral periapical radiographs were evaluated for the absence of any periapical pathosis. Confirmative pre-restorative checking of tooth Vitality was assessed using an electric pulp vitality tester and cold test. One hundred and eight eligible teeth were enrolled after screening one hundred and twenty-five patients.

For ethical and legal issues, patients who agreed to participate in the research signed an informed consent that explained the procedures, follow-up period, and consequences that may happen in addition to the benefits and possible adverse effects of the application.

### Eligibility criteria

The included patients in this study were selected to be aged 15:40 years old, displaying mechanical (traumatic) pulp exposure while receiving treatment for active deep carious lesions, diagnosed as reversible pulpitis. Moreover, teeth inclusion criteria were tooth with a normal non-lingering positive reaction to thermal stimulation in a cold test, which is indicative of pulp vitality, normal periapical region, and closed apex are depicted in the periapical radiograph, and exposures that are within an estimated size range of 1–2 mm^2^ exhibiting controlled light red bleeding. However, patient exclusion criteria were: Immune-compromised patients or those with systemic medical disorders, those unable to return for 2nd restorative session or follow-up appointments, participants with mental and physical disabilities, pregnant females, and patients with a recent history of antibiotic and analgesic administration. As for teeth exclusion, the following criteria were eliminated: Teeth with spontaneous pain or sensitivity(discomfort) to percussion, symptoms of irreversible pulpitis, teeth with periodontal lesions, mobility beyond physiological range, Sinus opening, or abscessed tooth vestibular swelling, radiographic examination revealed, interrupted or broken lamina dura, widened periodontal ligament space, periapical radiolucency, internal or external root resorption or calcified canals, pulp bleeding that could not be controlled within 10 min of using diluted sodium hypochlorite, teeth that were not isolated by the rubber dam at the time of exposure, teeth with deep subgingival margins, or did not erupt sufficiently to allow isolation with a rubber dam, and non-restorable teeth or Teeth with insufficient tooth structure for direct restoration or when intracanal means of retention are needed.

### Randomization, allocation and grouping

**Fig. 1:**
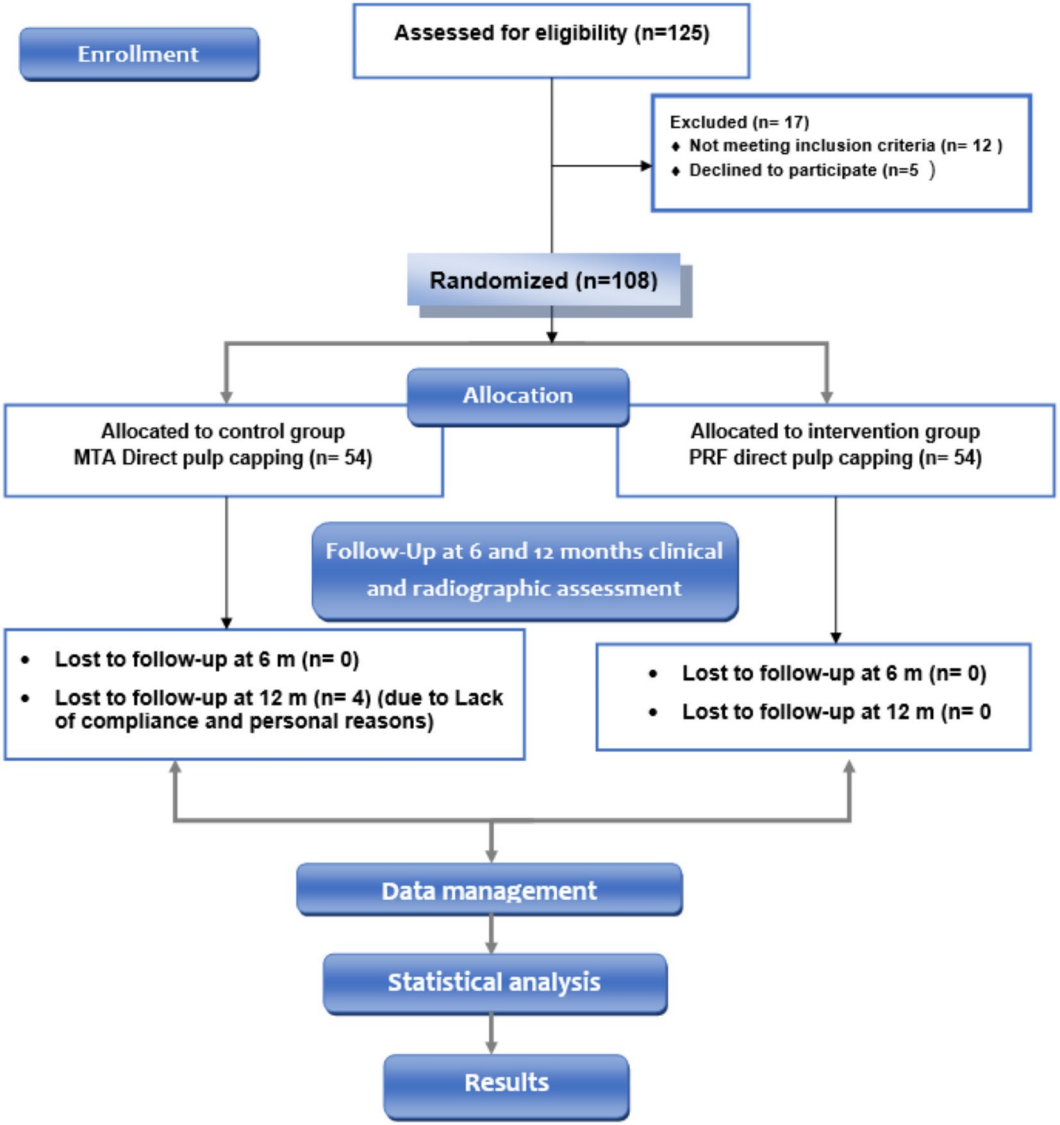
Consort flow diagram.

Simple randomization was performed utilizing a computerized sequence generator (www.random.org). According to a checklist done by a dentist other than the researcher, from 125 assessed patients, 108 patients with 108 teeth having exposed pulp during caries management were included in the study, 54 teeth for intervention (teeth to be treated with application of Platelet Rich Fibrin (PRF) directly over exposed pulp followed by MTA application), and 54 teeth in the comparator group (teeth were treated with direct application of MTA over exposed pulp). The flow chart of the patients through the study followed the CONSORT flow diagram is presented in Figure 1.

Each participant selected a random number from an opaque sealed envelope that was signed by the patient and the supervisor. the randomization list was kept in a secure location to enssure the total absence of any conflict from the operator.

### Blinding

The patient, the outcome assessors, and the statistician were blinded to the treatment group. However, blinding of the operator was impossible because of the difference in operative and application procedures between the control and intervention groups.

### Materials used in the study

The list of armamentariums and materials’ names, their descriptions/ Composition, Serial numbers, Lot numbers, manufacturers` or suppliers` names and websites were fully described in Table 1.

**Table 1:** List of armamentariums and materials’ ’names, their descriptions/ Composition, Serial numbers, Lot numbers, manufacturers` or suppliers` names and websites.

Armamentariums’ Names	Description/ Composition	Serial no./ LOT NO.	Manufacturer or supplier name and Website
Bench-top Centrifuge 800D	Blood biochemical qualitative analysis device to separate liquids from solids or different densities of fluids by applying a specific, consistent force to a specimen using centrifugal force, by spinning the fluid quickly inside a container.	**----------**	Biofield medical, China. supplied by DELTA lab company
IVAC vacutainer tubes plain no additive	For blood collection and transport, 5 ml glass tube with a plastic red cap to perform selected chemistry tests.	**LOT**: **2002Y121**	ElDawlia-Ico Med. Abu-Tig, Assiut-Egypt. https://medical-ico.com/
Nic tone Rubber dam sheets	100% NATURAL LATEX Rubber dam sheets powder-free, medium. 15 cm x 15 cm (6 × 6)	**LOT: 010222016**	MDC dental, Zapopan, Jalisco, México. https://www.mdcdental.com/our-brands/nictone/
Composite applicators (SD Innovation Magical Composite kit)	Metal composite applicator	------------	SD innovations, Pakistan
Spiral finishing and polishing wheels (sof-lex).	The spirals are made with either aluminum oxide or diamond particles impregnated in a thermoplastic elastomer. Sof-lex spiral Finishing and polishing wheels are 2 step system adapted on a mandrel. (Beige and pinkish diamond-impregnated one)	**N508795** **N508796**	3 M ESPE, St. Paul, MN, USA http://www.3MESPE.com
Electric pulp tester Denjoy DY310	Pulp tester is a device to examine the sensibility of dental pulp using electrical stimulation. Device supplied with (Control part, Test electrode, Test cable, Stainless hook).	**Lot: DY201202**	Denjoy Dental Co., Ltd., Changsha City, Hunan Province, China. https://www.denjoy.cn/
Cold pulp test (Endo ice)	Sensibility test spray with a minty scent. Bottle with 200 mL, Temperature − 50 °C Composition: Deodorized butane, ethanol, sodium benzoate, demineralized water, essence of menthol.	**LOT: 10,989** **Ref.: 0103001001**	Maquira Dental Products, Maringá, PR, Brazil) https://maquira.com.br/en/
Cone beam computed tomography (CBCT) Carestream CS 9600		**-------------**	**Rochester**,** New York**, **USA** http://www.carestream.com/
Acteon X-Mind DC intraoral X-Ray			Acteon group company, Mérignac, France. https://www.acteongroup.com/en/
Materials	**Composition**	**Serial no./ LOT NO.**	**Manufacturer or supplier name and Website**
Etchant: Acid gel Scotchbond™ Universal Etchant	-34% phosphoric acid by weight, pH of approximately 0.1. • Water • Orthophosphoric acid • Synthetic amorphous silica, fumed, crystalline free • Poly(oxy-1,2-ethanediyl), alphahydroomega-hydroxy-ethane-1,2-diol, ethoxylated	**7,954,387**	3 M Deutschland Gmbh, Neuss, Germany. https://www.3 m.com/3M/en_US/p/c/dental-orthodontics/
Conventional resin composite restoration (nanofilled) (Filtek Z350 XT Nano-hybrid resin composite (enamel, dentin AND body shades)	♣ **Polymeric matrix**: Bis-GMA, UDMA, TEGDMA, PEGDMA, Bis-EMA, ♣ **Filler**: Silica (20 nm nonagglomerated/ aggregated), zirconia (4–11 nm nonagglomerated/aggregated and agglomerated), clusters of zirconia/ silica aggregated particles (20 nm silica particles combined with 4–11 nm zirconia)	**Enamel**: NA57001 **Body**: -NA43367 -NE36053 **Dentin**: NA63401	3 M ESPE, St. Paul, MN, USA https://www.3 m.com/3M/en_US/p/c/dental-orthodontics/
Single Bond Universal Adhesive	Mild (pH = 2.7) 10-MDP Phosphate Monomer, dimethacrylate resins, HEMA, methacrylate-modified polyalkenoic acid copolymer (Vitrebond Copolymer), filler, ethanol, water, initiators, silane	**Lot: 20408B**	3 M Deutschland Gmbh, Neuss, Germany. https://www.3 m.com/3M/en_US/p/c/dental-orthodontics/
Mineral trioxide aggregate White MTA-Angelus^®^	Powder: Tricalcium silicate, dicalcium silicate, tricalcium aluminate, calcium oxide, plus Calcium Tungstate Liquid: Distilled water	**102,658**	(Angelus, Londrina, PR, Brazil) https://angelusdental.com/
Resin-modified glass ionomer liner Vitrebond	Powder: fluoroaluminosilicate glass powder with SiO2, AlF3, ZnO, SrO, cryolite, NH4F, MgO, and P2O5; diphenyliodonium chloride liquid: modified polyacrylic acid with pendant methacrylate groups, HEMA, water, and photoinitiator	**NA83270** **NA77150**	3 M-ESPE, St. Paul, MN, USA
Temporary filling Coltsol F	Zinc oxide, zinc sulfate monohydrate, calcium sulfate hemihydrate, diatomaceous earth, ethylene-vinyl acetate resin, natrium fluoride, and peppermint aroma.	**J33572**	Coltene/Whaledent AG, Altstätten, Switzerland. https://www.coltene.com/

### Carious lesion management

Before patient enrolment in the study, a thorough medical history, clinical and radiologic examination were reviewed with the operating student and recorded. All clinical procedures were performed by the principal investigator under loupes magnification of X5 and a working distance of 420 mm (ergovision, China). Sharp spoon excavators (Dentsply Maillefer, Ballaigues, Switzerland) were used to remove any carious dentin, if present, in areas distant from the site of exposure. Disinfection and hemostasis of the exposed pulp were performed with 1.25% sodium hypochlorite NAOCL (JK-dental, Egypt) by diluting 5% sodium hypochlorite with water in a 1:3 ratio using a plastic syringe. cotton pellet soaked in the solution was left in the cavity for 10 min to achieve adequate disinfection and pulpal hemostasis. If bleeding persisted beyond this, the tooth was excluded. Afterward, the tooth was rinsed using cotton moistened with sterile saline (Normal Saline solution, Otsuka, Cairo, Egypt) to remove excess NAOCL and dried with a dry cotton pellet.

following hemostasis, the eligible patients were randomly distributed into two equal groups, with 54 patients per group, according to test materials applied over exposed pulps where in the comparator group MTA was applied directly to cap the exposed pulp, and in the intervention group, pre-application of Platelet Rich Fibrin (PRF) was implemented followed by MTA.

### **Intervention group**

In this group, Platelet Rich Fibrin (PRF) preparation was accomplished according to the protocol developed by Choukroun et al. by drawing 5 ml of blood from the patient’s antecubital (forearm) vein and transferring them into a test tube devoid of any anticoagulant to be centrifuged using a table centrifuge (800D, Biofield medical, China) which has a rotor radius of nearly 10 cm and set to operate for ten minutes at 3000 RPM thus giving relative centrifugal force (RCF) of ≈ 1007 *×g*^43^. Following centrifugation, the subsequent three layers evolved: Acellular platelet-poor plasma at the top of the tube; Structured Fibrin clot (Platelet Rich Fibrin (PRF)) in the middle of the tube; and RBCs at the bottom of the tube^44^.

**Fig. 2:**
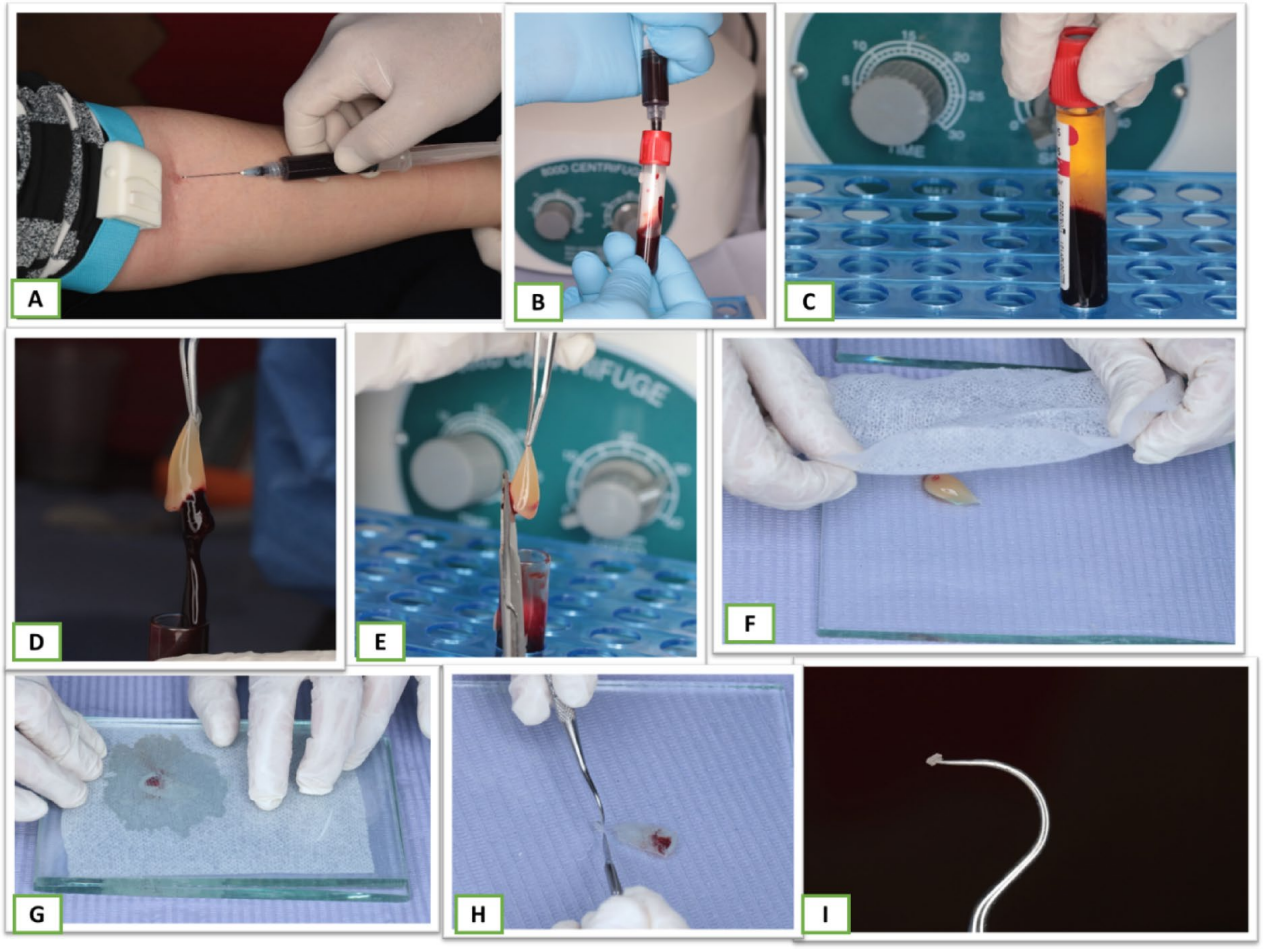
PRF preparation where (**A**) drawing 5 ml blood from the patient’s forearm. (**B**) transferring blood to plain test tube. (**C**) centrifuged blood sample. (**d**) grasped PRF from middle layer. (**E**) separating PRF from red corpuscle base layer. (**F**-**G**) applying PRF gel on glass slab followed by squeezing to drive out fluid. (**H**) cutting PRF membrane into small piece of 1 mm. (**I**) carrying PRF using tine of probe explorer.

**Fig. 3:**
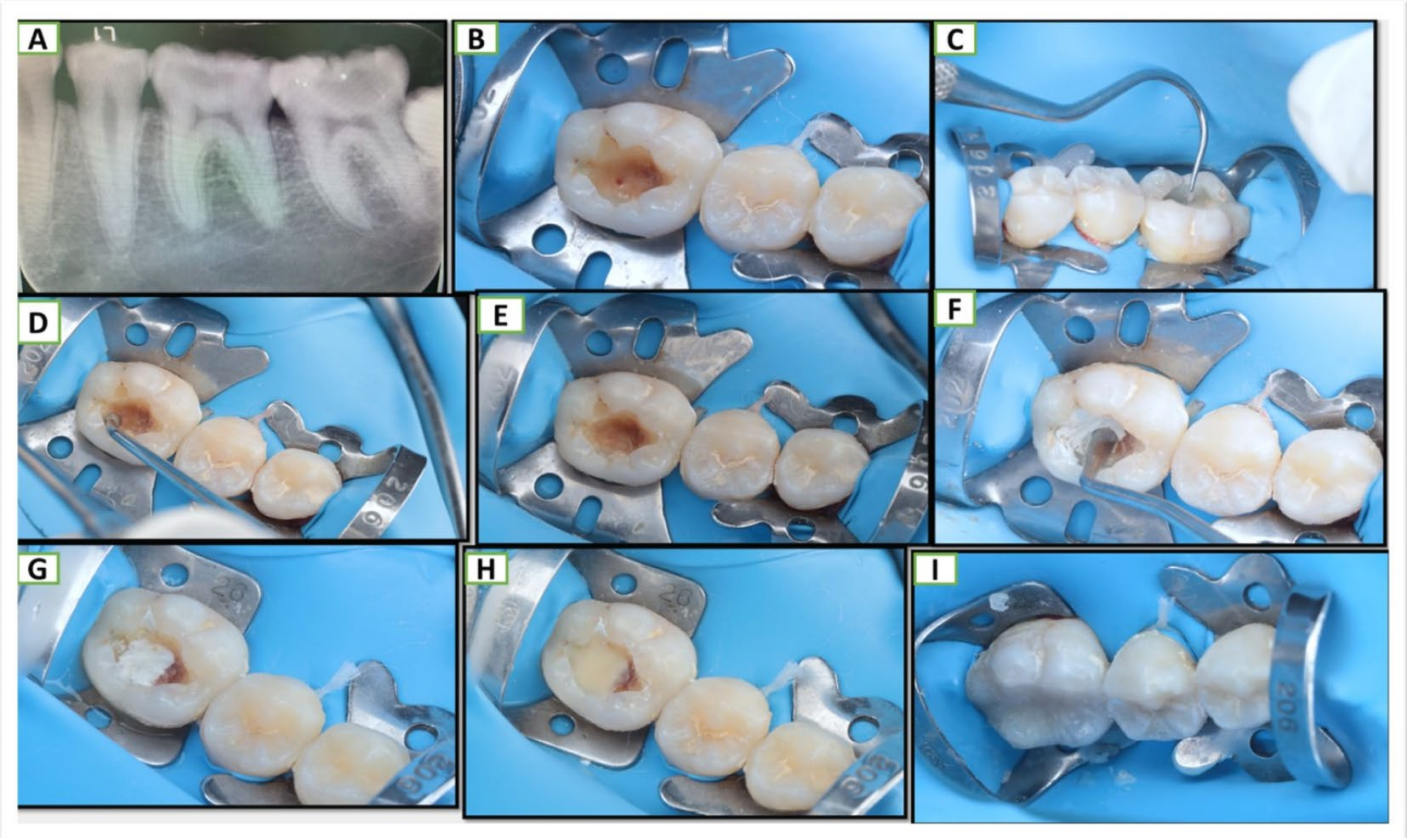
Sequence of pulp exposure capping procedure (**A**) preoperative periapical radiograph. (**B**) traumatic pulp exposure in lower first molar. (**C**) applying PRF with the tine of the probe explorer. (**D**) adapting PRF over-exposure site. (**E**) PRF covering exposure site. (**F**) applying MTA over the area of exposure. (**G**) fully set MTA. (**H**) RMGI liner over MTA. (**I**) composite restoration builds-Up.

The tip of a Sterile tweezer was gently placed into the blood tube to pick up a fibrin clot which was cut by a scissor. In order to extract the fluids contained in the fibrin matrix and create an extremely resilient autologous fibrin membrane, the Platelet Rich Fibrin (PRF) clot was pressed between a sterile dry gauge and squeezed gently between 2 glass slabs. Using a scalpel, Platelet Rich Fibrin (PRF) was cut into small pieces of 1 mm to be brought and placed over the site of exposure with the help of the tine of probe explorer Fig. 2. After that, a ball burnisher was used to ensure well-adapted Platelet Rich Fibrin (PRF) over the exposure site. Following the Platelet Rich Fibrin (PRF) application a layer of MTA (Angelus, Brazil) was mixed following the manufacturer’s instructions with a powder: liquid ratio of 1:1 using a stainless-steel spatula on a sterile glass slab. afterward, an amalgam carrier was implemented to carry and apply the mixture over the exposure site in a ≈ 1.5–2 mm thickness^37,45,46^ thereafter a condenser was used to gently adapt it Fig. 3.

### Comparator group

***In This group***, MTA (Angelus, Brazil) was mixed following the manufacturer’s instructions in the same manner as in the intervention group and applied directly over the exposed pulp without prior Platelet Rich Fibrin (PRF) application.

### Temporization phase

Then In both groups, MTA was topped with a cotton pellet dipped in normal saline, and the tooth was temporarily restored with Coltosol F(Coltène/Whaledent, Switzerland), and left for 3:7 days to ensure the complete setting of MTA before final restoration^23,42,47^.

### Restorative procedures

After 3: 7 days, temporary restoration was checked for continuity. Patients’ pulp vitality was ascertained using sensibility tests (electric and cold tests), as well as their tooth mobility, periodontal probing depth, and percussion sensitivity. Pre-restorative checking of occlusion and articulating point was performed using articulating paper. Under local anesthesia (4% Articane 1:100,000 Epinephrine, Septodont, France) and rubber dam isolation (Nictone, MDC dental, Zapopan, Jalisco, Mexico) temporary filling material was removed using a finishing diamond stone and excavator.

In both groups, Cavity was washed and dried with sterile cotton swabs to receive RMGI liner Vitrebond (3 M-ESPE, St. Paul, MN, USA). which was mixed according to the manufacturer’s instruction, applied over MTA with a ball burnisher, and light cured for 20 s, thereafter the cavity walls were finished with finishing stone. Selective enamel etching was performed with phosphoric acid for 20 s. It was then rinsed off and dried. This was followed by the application of the bonding agent (Single bond universal,3 M oral care, USA)), which was light-cured for 20 s. Enamel, dentin, and body shades of Nano-filled resin composite Z350 XT (3 M oral care) were placed into the cavity incrementally; according to the matching shade selected before rubber dam isolation; and light cured for 40 s using a portable hand-held light curing unit. Fig. 3(h-I).

### Finishing and polishing

Occlusion was checked for any premature contact to be adjusted using articulating paper. Afterward, Finishing and Polishing of the restoration was performed utilizing flame and tapered finishing stone from (MANI, Japan) under water coolant followed by 2 step Sof-lex spiral Finishing and polishing wheels ((3 M Espe, 3 M/ESPE, St. Paul, MN, USA), eventually polishing using a sequential Kenda polishing kit (Kenda, Liechtenstein, Germany)and Direct Dia Polishing Paste diamond polishing paste was done ( Shofu, Tokyo, Japan).

### Outcomes assessment

One month before the beginning of the assessment appointments, two expert assessors were involved in clinical assessment, and two other experts in the radiology department were involved in radiographic assessment. The assessors underwent calibration sessions until a high degree of agreement was reached. Such calibration was calculated on 10 patients not included in the trial. In case of disagreement extended in-depth discussion was done till reaching consensus. Cohen’s Kappa test tested Intra and inter-assessor agreement, which was 85% after the calibration sessions.

The two professional examiners performed the clinical evaluation under complete blindness. The restorations were coded. so, unawareness of the patients’ group ensured.

Patients in all groups were recalled for clinical and radiographic examinations after 6 and 12 months of clinical treatment.

### Vitality via sensibility tests

Pulp sensibility was assessed during follow-up using thermal cold testing (Endo Ice, maquira) and an electric pulp tester (Denjoy DY310). The response of the treated tooth to thermal pulp testing was compared to the control tooth (sound contralateral and opposite teeth) to determine the intensity as 1, a normal positive reaction (pain lasting up to 10 s); 2, an exaggerated positive reaction; 3, no reaction. and duration (lingering > 10 s or not)^48,49^ .

In Electric pulp testing (EPT) Patients experience a gentle, pulsed stimulus or a “tingling” sensation once the increasing voltage reaches the pain threshold which varies between patients, thus patients are informed to raise their hands when they reach this feeling for reading to be recorded. Three repetitions of the electrical pulp test were conducted to guarantee inter/intra-examiner reliability. The contralateral tooth in the same arch was used for comparison measurements^48,50^.

### History of pain

The patients were asked whether they had experienced any pain or discomfort during the follow-up period. A verbal pain intensity scale was used to describe felt pain that comprises absence, weak, moderate, and strong^42^. To evaluate the duration of the pain, the following categories were applied: 0, no pain; 1, symptoms for a single day; 2, symptoms for 2:7 days; and 3, symptoms for more than seven days^51^.

### Pain on percussion

Participant’s response to percussion whether a positive response (i.e., pain) or negative (i.e., no pain or discomfort) was recorded.

### Clinical or radiographic signs and /or symptoms of pulp necrosis or apical periodontitis

Treatment was considered successful if there wasn’t any sign or symptom of pulp necrosis (abscess, swelling, widening of periodontal ligament space, loss of lamina dura, internal or external resorption, and periapical or furcal pathosis). Standardized digital periapical radiographs were performed at baseline T0 (immediately after restoration), after 6 months T1 and after 12 months T2.

### Digital radiographic procedure

Digital intraoral imaging system which comprises Digora Optime DXR-50 001 (Soredex-Finland) digital intraoral imaging scanner and Size 2 photostimulable phosphor plate (Soredex Corp., Tuusula, Finland) was used. Images were displayed and analyzed on the computer monitor.

Standardized Radiographic exposure was performed utilizing a posterior parallel kit film holder (FPS 3000 film positioning system) and customized acrylic bit block to guarantee similar receptor placement and precise relocating of the bite block at baseline and follow up. The imaging plate was stabilized onto the film holder and all the assembly was inserted in the patient’s mouth and the plastic aiming ring was fixed flush ended with the round end of the long cone.

Radiographic exposure was performed using the Acteon X-Mind DC intraoral X-Ray (France) with the following parameters: 70kVp, 7 mA, and exposure time 0.12 s. Exposure parameters were fixed for all patients at the baseline and during the follow-up.

### Radiographic image analysis

An evaluation of periapical radiographs was carried out using the Periapical Index (PAI) which was created by Orstavik et al. and is regarded as the most widely used index for periapical health assessment^52^. The scores used in this system were:

**Table:** 

Score 1:	Normal periapical structures.
**Score 2**:	Small changes in bone structures.
**Score 3**:	Change in bone structure with mineral loss
**Score 4**:	Periodontitis with well-defined radiolucent area.
**Score 5**:	Severe periodontitis with exacerbating features

Periapical radiographs at 6 months (T1) and 12 months (T2) were compared to the baseline (T0). each tooth’s periapical index score (PAI) was reported. The assessors arrived to an agreement for every tooth after seeing the periapical radiographs individually^50^.All the obtained findings were confirmed with the more accurate CBCT assessment performed at 12 months to minimize radiation exposure as possible.

**Fig. 4:**
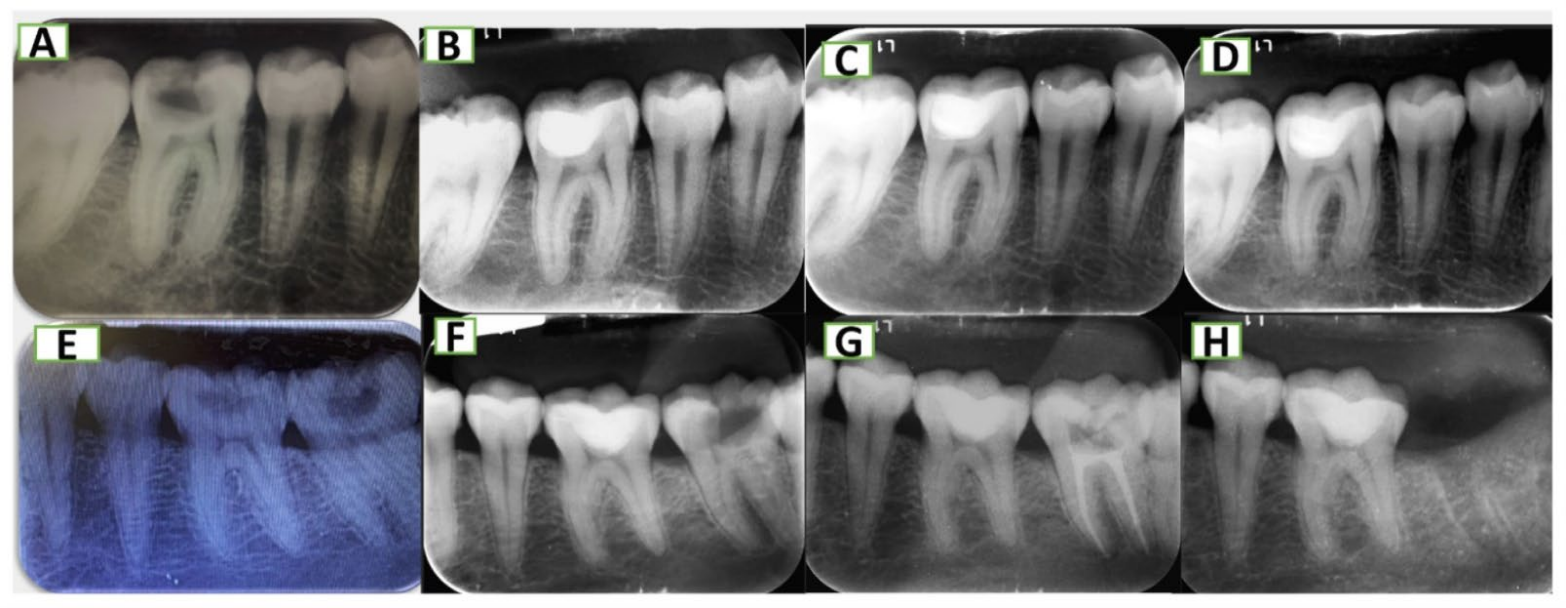
Radiographic assessment of periapical area (**A**, **B**, **C** and **D**) periapical radiographs for MTA capped lower right first molar preoperatively, at baseline, 6 months, and 12 months respectively, showing normal trabeculation of bone around the tooth with normal PDL space around roots. (**E**, **F**, **g** and **H**) preoperative, baseline, 6 months, and 12 months radiographs for lower left first molar capped with PRF and MTA showing normal bony architecture.

**Fig. 5:**
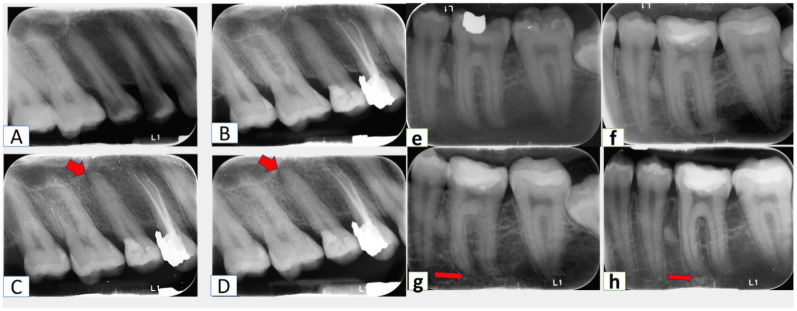
Failure of pulp capping treatment (a-b-c-d) in MTA group where (**A**) preoperative periapical radiograph. (**B**) baseline of pulp capping treatment with MTA in upper right second premolar with MOD cavity. (**C**) 6 months follow-up radiograph. (**D**) 12-month follow-up of MTA treatment, where arrows indicate periapical lesions. (e-f-g-h) PRF group where (**E**) preoperative periapical radiograph. (**F**) baseline of pulp capping treatment with PRF in lower left first molar with compound cavity. (**G**) 6 months follow up radiograph with score of 2. (**H**) 12-month follow up of PRF treatment score of 5.

In the majority of studies applying PAI index, the initially originated five categories (full scale) of the PAI scores divided into two dichotomies: PAI 1 and 2 (meaning “success” or “healthy”) and PAI 3–5 (meaning “failure” or “diseased”), And instructions while scoring was followed by outcome assessor. (Figs. 4 and 5). All the obtained findings were confirmed with CBCT.

Treatment was judged successful if the patient demonstrated no clinical symptoms with normal response to sensibility tests besides a PAI score of 1 or 2. However, Failure of the treatment was considered when at least one adverse clinical finding was present in the pulp sensibility test, pain on percussion, or claimed history of moderate or severe pain regardless of PAI score. Furthermore, the presence of a PAI score of 3 or above regardless of clinical symptoms was judged as a failure. All the obtained findings were confirmed with CBCT.

### Tomographic assessment of regenerative dentin formation

Tomographic assessment for the formation of dentin bridge over the exposure site (yes or no) was performed after 12 months(T2) through the submission of All participants for CBCT. CBCT images were acquired using a Carestream CS 9600 scanner (Carestream, New York, USA). A scout view was obtained and adjustments were made to ensure that all samples were correctly aligned in the scanner according to the adjustment light beam before acquisition.

The machine was operated at the following protocol and exposure parameters for all the scans of the study: Tube Voltage 120 kVp, 6.3 mA, Voxel size 75 μm, 19 s Scanning time, and 5 × 5 Field of view.

After acquisition, data were exported and transferred in DICOM (Digital Imaging Communication) format and downloaded via a Compact Disk (CD) to a personal computer for analysis by a blinded expert radiologist, where all Images DICOM format were imported into OnDemand 3d App software (Cybermed, South Korea) utilized for assessment. The images were examined on a separate computer with specific Radiology display to detect the various grey shades at the images. Images were examined at BARCO NiO monitor (Barco, Kortrijk, Belgium), with resolution of 3 MP, bit depth of 30 bit, and DICOM calibrated luminance of 600 cd/m².

**Fig. 6:**
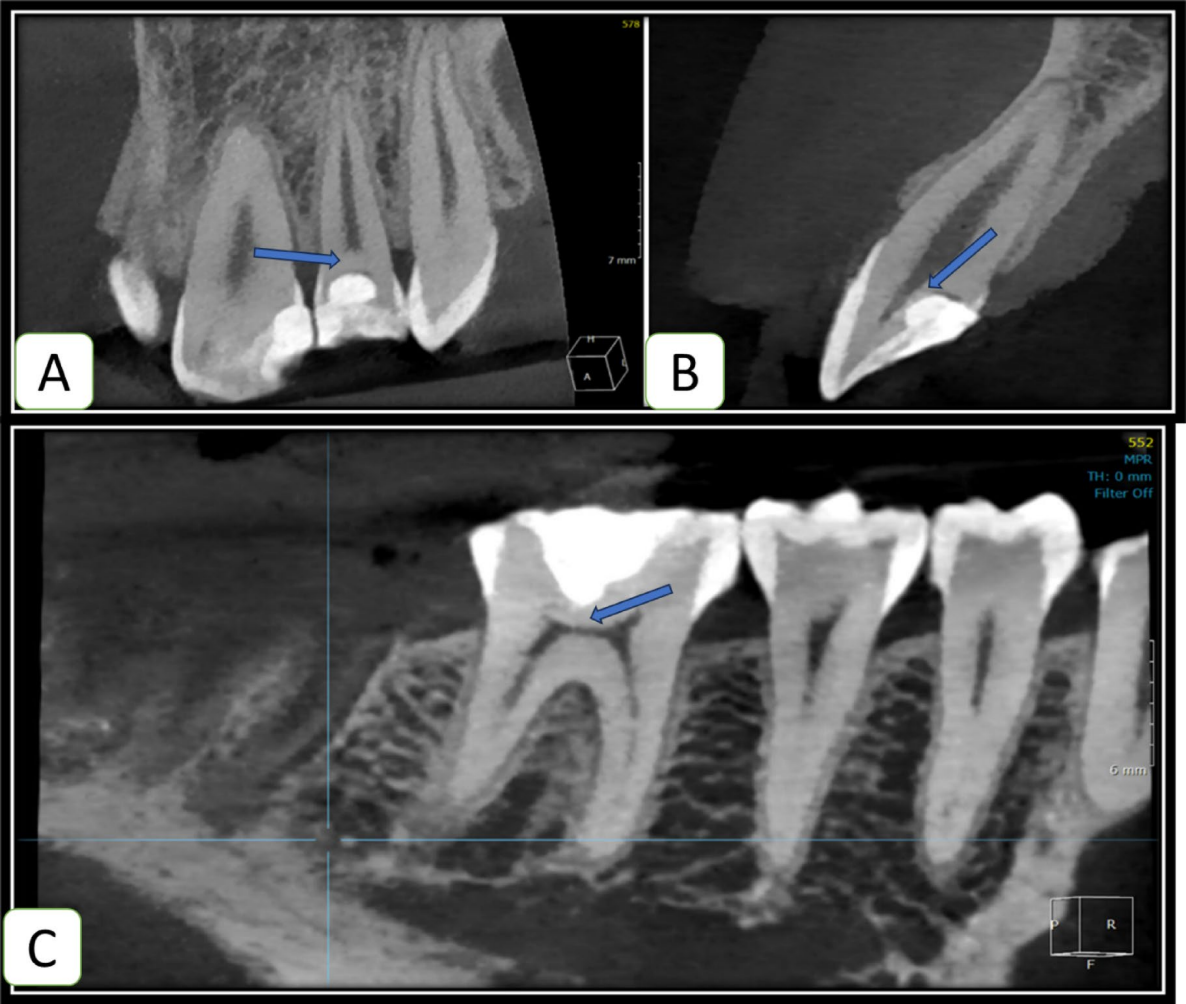
Tomographic evaluation where arrows showing dentin bridge formation (**A**-**B**) different views of MTA capped lateral incisor (**C**) PRF capped first molar as dedicated in OnDemand3D App.

**Fig. 7:**
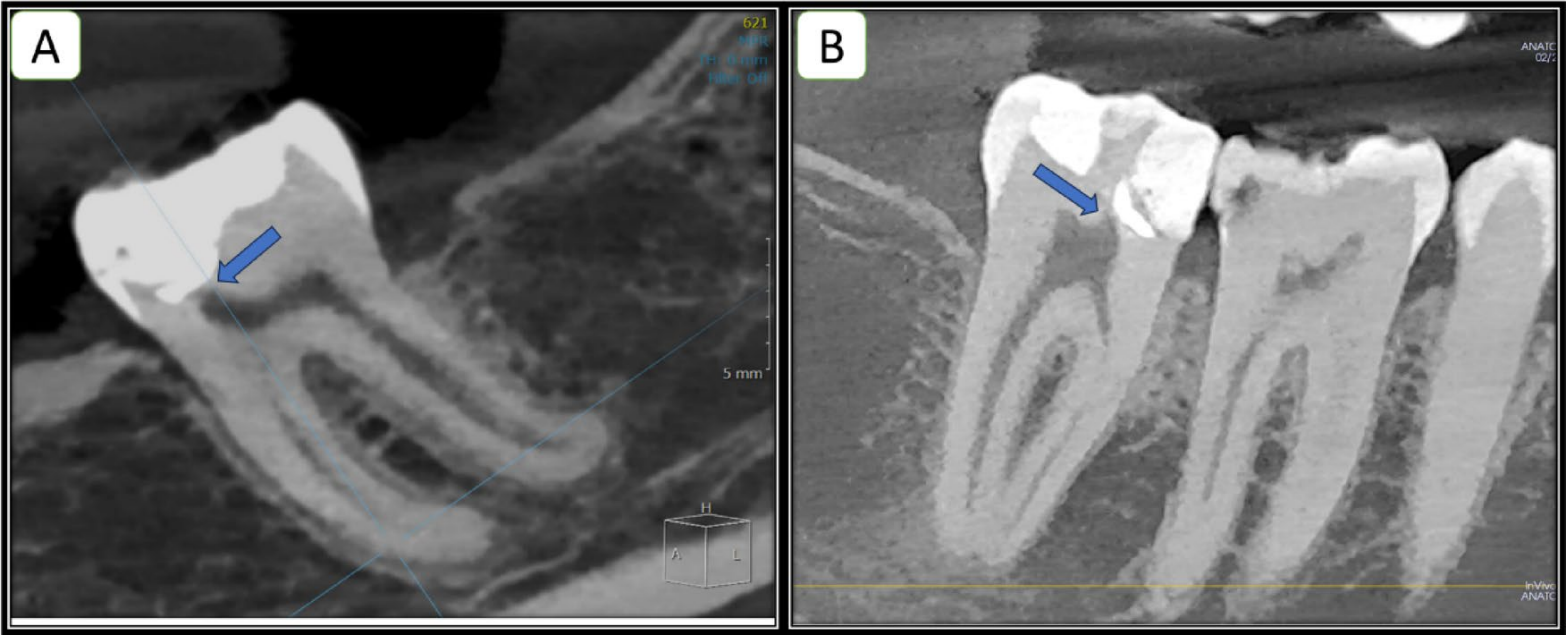
Tomographic evaluation showing the Absence of dentin bridge formation overexposure site as indicated by arrows (**A**): in the MTA group while (**B**) in the PRF group.

MPR view (Multi-Planar Reconstruction) view was used to assess dentin formation. Reference images from axial, coronal and sagittal views were moved to align with the long axis of the examined tooth, and the restorative material was allocated, then the detection of dentin formation was carried away at coronal and sagittal perspectives. (Figs. 6 and 7***)***

### Statistical analysis

Statistical analysis was performed with R statistical analysis software version 4.4.1 for Windows. Ordinal and categorical data were presented as frequency and percentage values. Categorical data were analysed using Fisher’s exact and McNemar’s tests for inter and intragroup comparisons, respectively. Ordinal data were analysed using Mann-Whitney and signed rank tests for intergroup and intragroup comparisons, respectively. Age data were presented as mean and standard deviation values. They were tested for normality using Shapiro-Wilk’s test. They were found to be normally distributed and were compared using an independent t-test. The Cox regression model was used to study the effect of different variables on survival. The Survival plot was made using the Kaplan–Meier estimator. The significance level was set at *p* < 0.05 within all tests.

## Results

108 patients submitted informed consent, participated in the trial, and received treatment as assigned. Table 2 illustrates the baseline demographic data of the participants including sex, age, and tooth type, and tooth position. Four patients did not attend 12 months follow-up appointment due to noncompliance or personal reasons and all of them belong to the MTA group. There was no significant difference between both groups regarding different demographics and baseline characteristics. Most treated teeth in both groups were in the posterior region and had class (II) lesions and most of them were in the posterior region.

**Table 2:** Intergroup and summary statistics for demographic data.

Parameter		MTA Group	PRF Group	*p*-value
**Gender** **[n (%)]**	**Male**	20 (37.04%)	23 (42.59%)	**0.694**
	**Female**	34 (62.96%)	31 (57.41%)	
**Age (Mean ± SD) (years)**		26.85 ± 8.94	24.63 ± 6.20	**0.136**
**Cavity type** **[n (%)]**	**Class (I)**	16 (29.63%)	21 (38.89%)	**0.603**
	**Class (II)**	25 (46.30%)	22 (40.74%)	
	**Class (III)**	13 (24.07%)	11 (20.37%)	
**Treated arch** **[n (%)]**	**Upper**	32 (59.26%)	27 (50.00%)	**0.440**
	**Lower**	22 (40.74%)	27 (50.00%)	
**Treated tooth** **[n (%)]**	**Anterior**	13 (24.07%)	11 (20.37%)	**0.817**
	**Posterior**	41 (75.93%)	43 (79.63%)	

After 6 months Findings of the overall treatment outcome revealed a significantly higher percentage of failed cases in the MTA group (*p* = 0.006). However, After 12 months there were four failed cases in each group with equal overall success of treatment of 92.59%(*n* = 50) (*p* = 1). Table 3.

**Table 3:** Inter, intragroup comparison, and summary statistics for treatment outcome.

Interval	Treatment outcome	*n* (%)		*p*-value
		MTA Group	PRF Group	
**6 months**	**Success**	46 (85.19%)	54 (100.00%)	0.006*
	**Failure**	8 (14.81%)	0 (0.00%)	
**12 months**	**Success**	50 (92.59%)	50 (92.59%)	**1**
	**Failure**	4 (7.41%)	4 (7.41%)	
**p-value**		**0.134**	**0.134**	

Table 4 represents inter-, intragroup comparisons and summary statistics for thermal pulp testing categorized as: overall thermal pulp testing response, exaggerated response, and lower intensity response. Results of pulp sensibility tests showed a negative response of 4 cases (7.41%) in the MTA group to the thermal cold test at both intervals, while all the cases in the Platelet Rich Fibrin (PRF) group responded positively to the cold test response. The difference between both groups was not statistically significant (*p* = 0.118). however, all treated cases in both groups responded positively to the electric pulp test at both intervals Table 4.

**Table 4:** Inter, intragroup comparisons and summary statistics for thermal pulp testing (overall thermal pulp testing response, exaggerated response to thermal pulp testing, lower intensity response).

Interval	Thermal pulp testing	*n* (%)		*p*-value	Exaggerated response to thermal pulp testing	*n* (%)		*p*-value	Lower intensity response	*n* (%)		*p*-value
		MTA Group	PRF Group			MTA	PRF Group			MTA group	PRF group	
**6 months**	**Negative**	4 (7.41%)	0 (0.00%)	**0.118**	**No**	54 (100.00%)	46 (85.19%)	0.006*	**No**	54 (100.00%)	54 (100.00%)	**NA**
	**Positive**	50 (92.59%)	54 (100.00%)		**Yes**	0 (0.00%)	8 (14.81%)		**Yes**	0 (0.00%)	0 (0.00%)	
**12 months**	**Negative**	4 (7.41%)	0 (0.00%)	**0.118**	**No**	45 (83.33%)	54 (100.00%)	0.003*	**No**	54 (100.00%)	50 (92.59%)	**0.118**
	**Positive**	50 (92.59%)	54 (100.00%)		**Yes**	9 (16.67%)	0 (0.00%)		**Yes**	0 (0.00%)	4 (7.41%)	
**p-value**		**1**	**NA**			0.003*	0.005*			**NA**	**0.072**	

History of Pain Intensity and Duration After 6 months 9 cases in the MTA group had weak pain, while 4 had moderate pain. In the Platelet Rich Fibrin (PRF) group, 14 cases had weak pain, and the difference between both groups was not statistically significant (*p* = 0.997). After 12 months, 5 cases in MTA group had weak pain, and the difference between both groups was statistically significant (*p* = 0.023). all reported pain lasted only for single day and within both groups, there was a significant reduction of the number of affected cases over time Table 5. None of the treated cases in both groups reported tenderness to percussion in both intervals neither at 6 months nor at 12 months.

**Table 5:** Inter, intragroup comparisons and summary statistics for pain intensity and duration.

Interval	Pain intensity	*n* (%)		*p*-value	Pain duration	*n* (%)		
		MTA Group	PRF Group			MTA Group	PRF Group	
**6 months**	**Absence**	41 (75.93%)	40 (74.07%)	**0.997**	**No pain**	41 (75.93%)	40 (74.07%)	**0.828s**
	**Weak**	9 (16.67%)	14 (25.93%)		**Single day**	13 (24.07%)	14 (25.93%)	
	**Moderate**	4 (7.41%)	0 (0.00%)		**2–7 days**	0 (0.00%)	0 (0.00%)	
	**Severe**	0 (0.00%)	0 (0.00%)		**More than 7 days**	0 (0.00%)	0 (0.00%)	
**12 months**	**Absence**	49 (90.74%)	54 (100.00%)	0.023*	**No pain**	49 (90.74%)	54 (100.00%)	0.023*
	**Weak**	5 (9.26%)	0 (0.00%)		**Single day**	5 (9.26%)	0 (0.00%)	
	**Moderate**	0 (0.00%)	0 (0.00%)		**2–7 days**	0 (0.00%)	0 (0.00%)	
	**Severe**	0 (0.00%)	0 (0.00%)		**More than 7 days**	0 (0.00%)	0 (0.00%)	
**p-value**		0.012*	< 0.001*		**p-value**	0.006*	< 0.001*	

### Radiographic evaluation

By using periapical index scores (PAI) to categorize bony changes and periapical pathosis. After 6 months,4 cases (7.4%) in the MTA group scored 2 due to widening in the PDL space without loss of lamina dura continuity compared to 12 (22.2%) cases in the Platelet Rich Fibrin (PRF), Score 2 was considered within healthy and success criteria^52,53^. However, 4 failed cases received a score of 4 owing to reporting periodontitis with a well-defined radiolucent area all of them in the MTA group without statistically significant difference(*p* = 0.118) between both groups. After 12 months, a further failure was detected in four cases within the Platelet Rich Fibrin (PRF) group, with two of them receiving a score of 3 where larger and more irregular widening of PDL space was observed at the apical foramen area with evident demineralization and the other two cases received a score of 5 with statistically insignificant differences in both groups as 4 cases were failed in each group reporting 92.6% radiographic success of both groups Table 6.

**Table 6:** Inter, intragroup comparisons and summary statistics for radiographic outcome and PAI index.

Interval	PAI index	*n* (%)		*p*-value	Radiographic outcome	*n* (%)		*p*-value
		MTA Group	PRF Group			MTA Group	PRF Group	
**6 months**	**Score (1)**	46 (85.19%)	42 (77.78%)	**0.4475**	**Success**	50 (92.59%)	54 (100.00%)	**0.118**
	**Score (2)**	4 (7.41%)	12 (22.22%)					
	**Score (3)**	0 (0.00%)	0 (0.00%)		**Failure**	4 (7.41%)	0 (0.00%)	
	**Score (4)**	4 (7.41%)	0 (0.00%)					
	**Score (5)**	0 (0.00%)	0 (0.00%)					
**12 months**	**Score (1)**	46 (85.19%)	42 (77.78%)	**0.366ns**	**Success**	50 (92.59%)	50 (92.59%)	**1ns**
	**Score (2)**	4 (7.41%)	8 (14.81%)					
	**Score (3)**	0 (0.00%)	2 (3.70%)		**Failure**	4 (7.41%)	4 (7.41%)	
	**Score (4)**	4 (7.41%)	0 (0.00%)					
	**Score (5)**	0 (0.00%)	2 (3.70%)					
**p-value**		**1ns**	**0.095ns**			**1ns**	**0.072ns**	

### CBCT evaluation

Results of tomographic evaluation were correlated with periapical radiographs at 12 months follow-up, each root was inspected for any pathological alterations. There were no signs of furcal pathosis, root resorptions, or root canal obliterations in any of the treated teeth in both groups. Concerning the assessment of dentin bridge formation, it was formed in a significantly higher percentage of cases in the PRF group 92.59% (*n* = 50) compared to 48.15%(*n* = 26) in the MTA group (*p* < 0.001). Table 7.

**Table 7:** Intergroup comparison and summary statistics for dentine bridge formation.

Dentine bridge formation	n (%)		p-value
	MTA Group	PRF Group	
**No**	28 (51.85%)	4 (7.41%)	< 0.001*
**Yes**	26 (48.15%)	50 (92.59%)	

Results for survival regression analysis are presented in Table 8, where the cavity class and tooth type were excluded from the model as all the failed cases were in posterior teeth with compound cavities.

**Table 8:** Results for survival regression analysis.

Variable	Hazard ratio	95% Confidence interval		Tests statistic	*p*-value
		Lower	Upper		
**PRF Group**	0.34	0.08	1.45	**-1.46**	**0.146ns**
**Sex (female)**	0.41	0.12	1.42	**-1.41**	**0.157ns**
**Age**	0.98	0.89	1.07	**-0.46**	**0.643ns**
**Dentine bridge formation (yes)**	1.37	0.24	7.70	**0.35**	**0.724ns**

## Discussion

The current study was conducted to comparatively assess the clinical success and radiographic regenerative dentin formation of Platelet Rich Fibrin (PRF) and mineral trioxide aggregate when used as direct pulp capping agents.

The tested null hypothesis is partly accepted as there is no significant difference in success rate when Platelet Rich Fibrin (PRF) is applied prior to MTA in direct pulp capping compared with the application of MTA alone. However, it was partly rejected regarding regenerative dentin formation as newly formed dentin was more significant when Platelet Rich Fibrin (PRF) was applied prior to MTA.

In the current investigation, combining Platelet Rich Fibrin (PRF) with MTA was implemented to be compared to using MTA alone as a direct pulp capping agent to counteract the action of included toxic component in MTA with this biological scaffold thus preventing additional harm to the remaining odontoblasts besides boosting the differentiation of new odontoblasts through included growth factors^54^. This regenerative biological approach has been implemented in multiple pulpotomy and apexogensis studies^31,55–57^. Nevertheless, to the best of the authors’ knowledge and research, until the setting of this study protocol, there was no clinical trial investigating the role of Platelet Rich Fibrin (PRF) in direct pulp capping.

This study is double-blinded; the participants, outcome assessors and statistician were blinded to study group assignments to minimize the risk of performance and detection bias. Besides, concealment of sample allocation and simple computer-generated randomization were implemented to prevent selection bias thus giving each participant an equal chance of being assigned to a certain group for ensuring equal distribution of potential confounding factors.

Considering a high level of intra and Inter-examiner agreement through training and calibration before starting the assessment procedures was mandatory in the present study to ensure standardized assessment, reliability, consistency, and reproducibility of the measured outcome.

Clinical and radiographic assessment of the treatment outcome and pulp vitality was implemented at 6 and 12 months of treatment. Tomographic evaluation via CBCT was performed at the end of 12 months follow-up only to confirm the health of the periapical tissue with a much more accurate tool and determine whether dentin had been formed over the exposure site or not^58^. Thereafter, giving a chance for adequate mineralized of newly formed dentinal tissue to be detected while reducing radiation exposure as much as possible^50,59^.

The findings of clinical and radiographic outcomes assessment in the present investigation revealed equal success rate in both treatment group (92.59%). As, four cases in the MTA group failed to respond to cold pulp sensibility testing at both follow-up intervals which was coincident with radiographic signs of treatment failure. However, the four failed cases at 12 months follow-up in the Platelet Rich Fibrin (PRF) group showed signs of pulp necrosis and periapical inflammation in periapical radiographs and CBCT despite lower intensity response to the cold test was reported.

Non-lingering exaggerated response to pulp test is considered an indication of reversible pulpitis^60^, it can also indicate a lowered patient threshold^48,49^ which necessitates further follow-up.

Regarding the findings of EPT, All the treated cases in both groups showed a positive response to the electric pulp tester, which may be attributed to the greater technique sensitivity of the test and the necessity of a dry field^61^. Sensibility pulp testing has a significant flaw not only for being a subjective method that relies on symptoms reported by the patient for disease diagnosis but it also measures a neuronal response rather than the vascular supply, which means that it only indirectly reveals the vitality condition of the pulp. As a result, false positive and false negative results are possible^49^.

Furthermore, Systematic review by **Mainkar & Kim in 2018** revealed that EPT exhibits diminished accuracy and low sensitivity, implying a reduced likelihood of accurately identifying nonvital teeth, while demonstrating high specificity, indicating an increased likelihood of correctly identifying vital teeth^62^, which could explain the obtained false positive results. A variety of clarifications for this error with EPT have been suggested including engaging with adjacent metallic restorations, Periodontal tissue degradation products from necrotic pulps and remains of inflammatory pulp tissues may induce sensory stimulation resulting in erroneous responses, moreover, a multi-rooted tooth may exhibit inflammatory pulp tissue in one canal, while the pulp chamber and another canal could be necrotic and infected^49,63^ thus contributing to the false positive results.

Conversely, Cold tests are less likely to produce false positive results because Cold stimulation cannot be achieved through fluids or necrotic pulp tissue, as it requires more live tissue in the tooth’s coronal area compared to an EPT. Thus, Studies recommended Employing a cold pulp test to identify nonvital teeth instead of EPT and Further attention to clinical and radiological findings was suggested if the tooth did not respond to the cold test but did respond to EPT^49,62^.

Regarding the history of pain reported by the patients within follow-up period. The current study regarded transient mild pain as a normal occurrence following pulp therapy, Nonetheless, the failure criteria in the aspect of pain history was considered for those exhibiting moderate or severe pain. This is according to a review by ***Pradittapong et al.***, who reported that Postoperative pain following VPT is a complicated and multifaceted issue that frequently defies simple explanation or prediction and there is a lack of studies focusing on postoperative pain following VPT. Despite this, the published findings reveal that postoperative pain is not experienced by all patients receiving VPT, and when they do, it is often mild with rare instances of moderate or severe pain and diminishes markedly within days. Nonetheless, the exact duration till the attainment of a pain-free condition remains uncertain^64^.

The mechanism of pain associated to pulp capping procedures is comparable to pulpitis-related pain and can be explained by the generated inflammatory and immunological mediators in reaction to microbial infections or injuries. These mediators encompass cytokines, prostaglandins, and neuropeptides. They activate nociceptors, sensory pain receptors, which facilitate the transmission of pain signals and pain perception^65^.

Raised overall success of treatment in the MTA group from 6 to 12 months is attributed to reported failed cases due to a history of moderate pain in the first 6 months of treatment which is diminished after that in the 12 months follow up which can be explained by the subjectivity of the outcome assessment.

The high success rate obtained with pulp capping treatment can be ascribed to MTA’s exceptional sealing capability which could also account for its reduced antibacterial efficacy; it can be explained by its post-setting expansion due to crystal growth and Precipitation of apatite-like crystals at the MTA-dentine interface and within dentine collagen fibrils enhancing push-out bond strength by creating chemical and mechanical connections between dentine and MTA. Those apatite-like crystals resulted from released calcium ions (Ca2+) interaction with Phosphorus from the tissue fluid to form calcium phosphate salts followed by hydration and calcium hydroxide is formed as a by-product that could potentially be able to seal the pulpal tissue^66^in contrast to pure CH, which dissolves with time (Dycal disappearing syndrome).

Multiple studies have indicated that MTA stimulates the expression and release of various elements and molecules with an active role in dentinogenesis, such as tenascin, a non-collagenous glycoprotein in the extracellular matrix that plays significant roles in migration, adhesion, cell proliferation, and mineralization^67^. As well as, MTA has been demonstrated to augment the expression of transcription factors, that are engaged in molecular interactions during dentinogenesis, such as Runx2. This may elucidate why it is more efficacious than CH in recruiting human dental pulp stem cells fostering their migration, adherence, and proliferation for the development of reparative hard tissues^19^.

Besides, MTA has been found to boost BMP-2 protein synthesis, which is pivotal in the mineralization and differentiation of odontoblasts^68^, in addition to triggering the synthesis of certain cytokines that facilitate optimal cell adhesion to the substance, and may contribute actively to the formation of a dentin bridge^69^.

**Laurent et al.**, discovered the role of CSM on the modulation of TGF-β1 secretion from dental pulp cells that stimulate and guide dental pulp Mesenchymal Stem Cells (MSCs) proliferation and differentiation into pre-odontoblast progenitor that migrates and differentiates into odontoblast-like cells forming Dentinoid tissue, which is indicative of a reparative process rather than regeneration^70^. Furthermore, unlike CH, MTA stimulates angiogenesis through vascular endothelial growth factor expression (VEGF), which enables tissue regeneration^19^.

By implementing a combination of Platelet Rich Fibrin (PRF) and calcium-based silicate materials in the current investigation a comparable success rate was obtained after 12 months of treatment which could be attributed to the potential role of fibrin membrane as a scaffold that may support tissue regeneration. In addition, Blood centrifugation has been reported to activate key growth factors including TGF-β, insulin-like growth factor, VEGF, platelet-derived growth factor (PDGF), and epidermal growth factor from platelet granules. These Growth factors appear to be slowly released during fibrin matrix resorption. This Liberation is supposed to last from one week to 28 days with peak levels potentially occurring around day 14^37,54^.

These released Growth factors are believed to play a pivotal role in the healing of wounds by Boosting angiogenesis and tissue regeneration^71^. Among them, the TGF-β growth factor may help mitigate the inflammatory reaction in the pulp while also serving as a crucial signalling molecule for odontoblastic differentiation. whereas angiogenic growth factors, such as VEGF and PDGF, are thought to promote the development of new capillaries at the healing site^72^ which could further facilitate tissue repair and regeneration.

**Huang et al.**, explained the ability of the Platelet Rich Fibrin (PRF) to induce cell proliferation by potentially upregulating the expression of Osteoprotegerin(OPG) and Alkaline phosphatase (ALP) to promote the growth and differentiation of DPCs^34^. Whereas ALP is a membrane-anchored glycoprotein, regarded as a hallmark of osteogenic differentiation that signifies the mineralization process and cell differentiation into odontoblasts. On the other side, OPG inhibits the differentiation of osteoclasts.

Moreover, the autogenic platelet concentrate encompasses a substantial quantity of slowly released release interleukin [IL] cytokines such as IL-1b, IL-4, IL-6 and TNFα signalling molecules and recruit stem cells from the^71^. Interleukins not only modulate pulpal inflammation but also IL-1b was reported to suppress the activation of MMP-1 and MMP-3^73^.

In consequence, by virtue of the confinement of leukocytes and a tiny lymphocyte within the Platelet Rich Fibrin (PRF) membrane that effectively modulates inflammatory and infectious processes, the Platelet Rich Fibrin (PRF) membrane frequently referred to as an immunological organizing node^55^ helped in the success of pulp capping treatment.

The major cause of direct pulp capping treatment failure in both the Platelet Rich Fibrin (PRF) and MTA groups can be the tricky, difficult and imprecise preoperative diagnosis of the pulp state as the only trustworthy and acceptable reference standard is the histological investigation which is feasible only for extracted teeth^74,75^. Additionally, the postponement of the final definitive restoration application may have a detrimental effect on the treatment and jeopardize the seal’s quality^74,76,77^ which could account for the finding that all failed cases belonged to extensive and MOD cavities.

Upon reviewing literature, there is a lack of clinical studies evaluating the efficacy of direct pulp capping with Platelet Rich Fibrin (PRF). The present study results are in agreement with the findings of the only published clinical trial agents performed by ***Shobana et al.***,*** in 2022*** that compared the clinical and radiographic success rate of Platelet Rich Fibrin (PRF), Platelet Rich Plasma (PRP) and MTA as pulp capping agents after randomizing 30 permanent posterior teeth among the three treatment groups, where clinical assessment revealed a statistically insignificant difference between the three capping agents with clinical success of 90%^37^.

***Tiwari et al.***,*** in 2024*** also reported no statistically significant difference between Platelet Rich Fibrin (PRF) and MTA when used in pulp capping of primary molars despite the higher success rate obtained in the group treated with Platelet Rich Fibrin (PRF) (82.6%) compared to (61.9%) in MTA group^76^.

However multiple studies investigated the clinical success rate of DPC with MTA among which the study conducted by ***Suhag et al.***^42^, reported a 93% success rate of treatment after 12 months which is nearly the same as the present study. However, a multicentre study by ***Hilton et al.***,*** in 2013*** reported 80.3% success of capping treatment after a median follow-up of nearly 15.6 months^13^, ***Kundzina et al.***,*** in 2017 also*** showed a 85% cumulative survival rate after 36 months of treatment^23^.

Regarding the secondary outcome, the evaluation of dentin bridge formation via CBCT, the Platelet Rich Fibrin (PRF) group showed a statistically significant difference with 92.6% of cases showing evidence of dentin bridge formation in comparison to 48.15% only of cases in the MTA group. The results of the formed dentin bridge are in agreement with the results of ***Shobana et al.***, reporting a significantly higher volume of dentine bridge formed by Platelet Rich Fibrin (PRF) compared to MTA^37^. It can be explained by the efficacy of MTA to cause dentin bridge through triggering biochemical pathways, despite Platelet Rich Fibrin (PRF) additionally supplying the required bioactive ingredients in a preformed ready-to-use form. Thereafter the tested hypothesis is partly accepted and partly rejected.

### Limitation of the study

This study has certain shortcomings as it can be conducted with extended follow-up durations on a larger sample size across various clinical settings and histo-morphometric analysis, which is the accepted method for evaluating dentin bridges, can be performed to corroborate the findings of the current research.

## Conclusion

Based on this trial’s findings, it could be concluded that direct pulp capping with the novel pulp capping agent, Platelet Rich Fibrin (PRF), exhibits a clinical and radiographic success rate comparable to that of MTA. However, CBCT analysis revealed that PRF seems to have a greater potential to induce dentin bridge development.

### Recommendations

Further investigations are required to analyze the quality and quantity of the formed dentin bridge when Platelet Rich Fibrin (PRF) is employed, along with studies to evaluate the impact and success of combining platelet concentrates with different materials when implemented in vital pulp therapy.

### Clinical relevance

Platelet Rich Fibrin (PRF) can be implemented as a direct pulp capping agent in forthcoming clinical applications.

## Data Availability

All data generated are included in the current manuscript and available upon reasonable request to the corresponding author.

## Abbreviations

PRF: Platelet rich fibrin
DPC: Direct pulp capping
VPT: Vital pulp therapy
CBCT: Cone beam computed tomography
MTA: Mineral trioxide aggregate
RPM.: revolutions per minute.
TGF-β: Transforming growth factor-beta
PDGF: platelet-derived growth factor
VEGF: vascular endothelial growth factor
TNF: Tumor necrosis factor
RUNX2: Runt-related transcription factor 2 (RUNX2)
CH, Ca(oh)_2_: Calcium hydroxide
IL: Interleukin
ESE: European Society of Endodontology
CSM: Calcium silicate cements
PDL: Periodontal ligament space
NAOCL: Sodium hypochlorite
EPT: Electric pulp test
